# *Comamonas thiooxydans* Expressing a Plasmid-Encoded IMP-1 Carbapenemase Isolated From Continuous Ambulatory Peritoneal Dialysis of an Inpatient in Japan

**DOI:** 10.3389/fmicb.2022.808993

**Published:** 2022-02-21

**Authors:** Yuki Suzuki, Ryuichi Nakano, Akiyo Nakano, Hikari Tasaki, Tomoko Asada, Saori Horiuchi, Kai Saito, Mako Watanabe, Yasumistu Nomura, Daisuke Kitagawa, Sang-Tae Lee, Koji Ui, Akira Koizumi, Yuji Nishihara, Takahiro Sekine, Ryuji Sakata, Miho Ogawa, Masahito Ohnishi, Kazuhiko Tsuruya, Kei Kasahara, Hisakazu Yano

**Affiliations:** ^1^Department of Microbiology and Infectious Diseases, Nara Medical University, Kashihara, Japan; ^2^Department of Nephrology, Nara Medical University, Kashihara, Japan; ^3^Central Clinical Laboratory, Nara Medical University, Kashihara, Japan; ^4^Center for Infectious Diseases, Nara Medical University, Kashihara, Japan; ^5^Department of Bacteriology, BML Inc., Kawagoe, Japan

**Keywords:** *Comamonas thiooxydans*, IMP-1, metallo-β-lactamase, plasmid-mediated, whole genome sequence

## Introduction

*Comamonas thiooxydans* is a Gram-negative, rod-shaped, glucose-non-fermentative bacteria. The genus *Comamonas* is present in multiple natural environments; it has been isolated from sulfur springs, the termite gut, and water in natural and industrial environments (Chou et al., 2007; Narayan et al., 2010; Zhang et al., 2013; Hatayama, 2014). Although there are a few reports describing infections caused by *Comamonas* spp., including intra-abdominal infections and bacteremia (Almuzara et al., 2013; Zhou et al., 2018), *C. thiooxydans* has rarely been associated with human infections in clinical settings, with only one case of urinary tract infection reported to date (Guo et al., 2021).

Antibiotic resistance, particularly to carbapenems, is a threat to global health. Infections caused by carbapenemase-producing Gram-negative bacteria have limited treatment options and have high mortality (Tzouvelekis et al., 2014). Carbapenemase genes are frequently located on plasmids and mobile genetic elements that can be transmitted between species (Ludden et al., 2017). Recently, carbapenemase-producing Gram-negative bacteria have been reported from multiple species (Endo et al., 2012; Bonomo et al., 2018; Suzuki et al., 2019).

In this study, we isolated carbapenem-resistant *C. thiooxydans* from an inpatient in a hospital in Japan and investigated its molecular characteristics by whole-genome sequencing (WGS).

## Bacterial Isolation and Antimicrobial Susceptibility

Carbapenem-resistant *Comamonas* sp. strain NR4028 was isolated from a patient undergoing continuous ambulatory peritoneal dialysis at Nara Medical University Hospital in 2019. The isolate was identified as *Comamonas teststeroni* by matrix-assisted laser desorption/ionization time-of-flight mass spectrometry (MALDI-TOF MS) using a Vitek MS system (bioMérieux, Marcy-l'Étoile, France). The antimicrobial susceptibility of multiple antimicrobial agents was determined using the agar dilution method (Clinical Laboratory Standards Institute, 2015), and quality control was performed using *Escherichia coli* ATCC 25922. The minimum inhibitory concentrations for the isolate were: ceftazidime 256 μg/ml, cefepime 64 μg/ml, imipenem 1 μg/ml, meropenem 16 μg/ml, levofloxacin 32 μg/ml, gentamycin 256 μg/ml, and colistin 2 μg/ml.

## WGS

Genomic DNA was extracted using QIAGEN Genomic-tip 500/G (Qiagen, Germany) and sequenced using MiSeq (Illumina, United States), MinION (Oxford Nanopore Technologies, United Kingdom), and Sanger sequencing. After read trimming and quality filtering, hybrid *de novo* assembly was performed using Unicycler v0.4.9 (Wick et al., 2017). The assembled sequences were annotated using DFAST v1.4.0 with standard settings (Tanizawa et al., 2018). For species identification, average nucleotide identity (ANI) analysis was performed using *C. testosteroni* ATCC 11996 (GenBank accession no. AHIL01000000), *C. testosteroni* TK102 (GenBank accession no. CP006704), *C. thiooxydans* ZDHYF418 (GenBank accession no. CP063057), *C. thiooxydans* PHE2-6 (GenBank accession no. LKFB01000000), and *C. thiooxydans* DSM 17888 (GenBank accession no. LIOM01000000) as the reference genome (https://www.ezbiocloud.net/tools/ani). ANI values were 94.44, 92.54, 98.49, 96.66, and 96.83%, respectively. It was indicated that NR4028 had the highest homology with *C. thiooxydans* strains, and the species was determined as *C. thiooxydans*. Acquired resistance genes were identified using ResFinder version 4.1 (Bortolaia et al., 2020) on the Center for Genomic Epidemiology (CGE) server (http://www.genomicepidemiology.org/). Comparison of the plasmid sequence of *C. thiooxydans* NR4028 was performed using BLASTn (https://blast.ncbi.nlm.nih.gov/Blast.cgi) and visualized with Easyfig version 2.2.5. (Sullivan et al., 2011).

**Figure 1:**
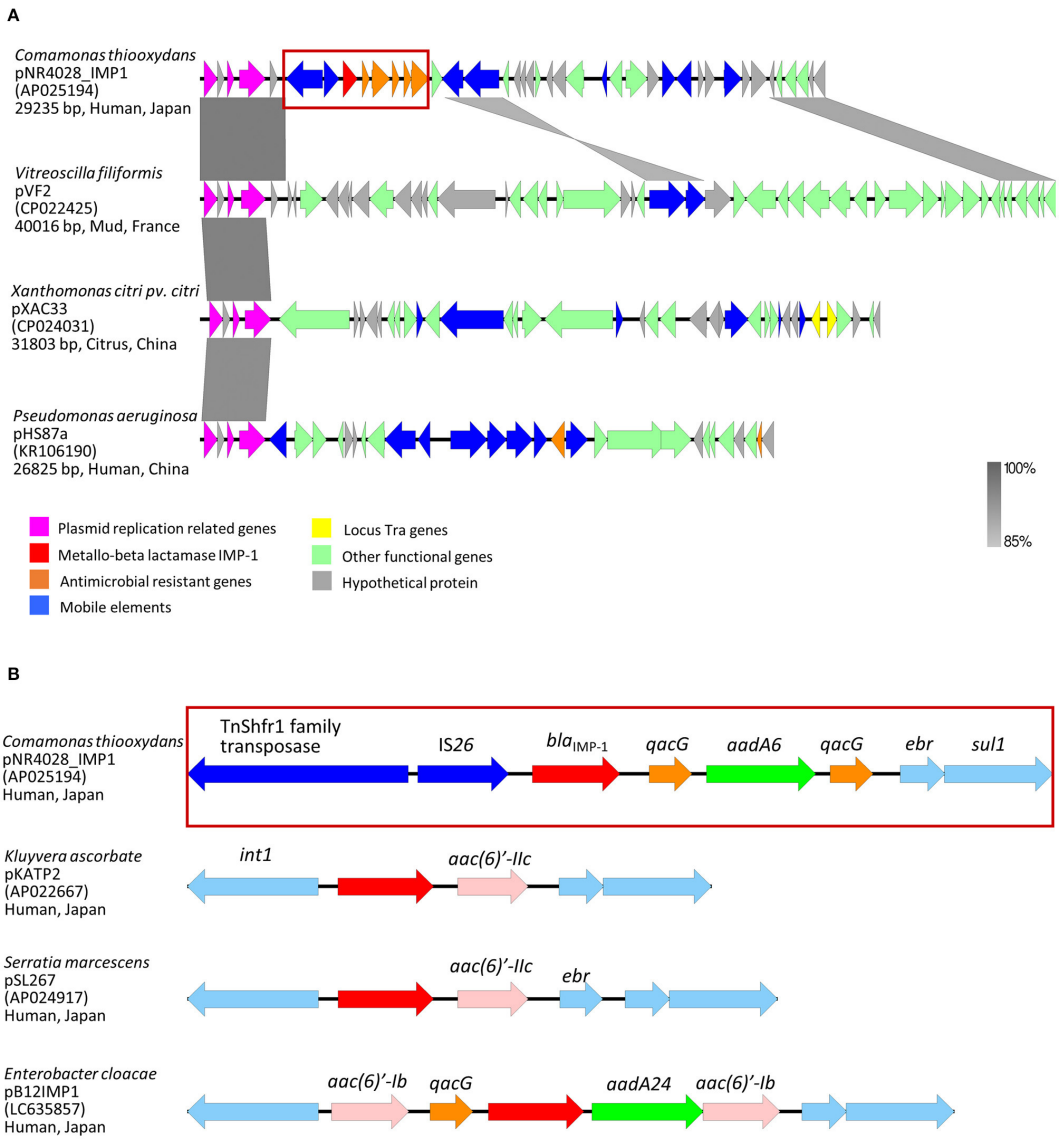
Genetic contexts of the IMP-1 encoding plasmid (pNR4028_IMP1) isolated from *Comamonas thiooxydans* NR4028. **(A)** Structural comparison of similar plasmid replicase in Gram-negative bacterial isolates. Regions with a homology are indicated by gray color. Genetic structure inside the red square frame is indicated in **(B)**, **(B)** Genetic environment of *bla*_IMP−1_ previously reported and NR4028. Genes were grouped and colored according to their predicted functions as indicated by the key. Arrows designate directions of transcription of genes and ORFs.

The genome sequence revealed three circular contigs with a total length of 5,620,102 bp and a G + C content of 61.2%. The genome of *C. thiooxydans* NR4028 consisted of one chromosome (5,588,008 bp; accession number AP025193) and two plasmids (29,235 bp, named pNR4028_IMP1; accession number AP025194, and 2,859 bp; accession number AP025195). ResFinder version 4.1 identified three antimicrobial resistance genes (*bla*_IMP−1_, *aadA6*, and *sul1*) on the plasmid pNR4028_IMP1. This IMP-1-encoding plasmid structure was different from the previously reported, and Figure 1 shows a map of pNR4028_IMP1.

## Conjugation Assay

To determine the transferability of the IMP-1 gene, we used the filter mating method, with *C. thiooxydans* NR4028 as the donor and sodium azide-resistant *E. coli* J53 and rifampicin-resistant *Pseudomonas aeruginosa* PAO1 as the recipient, as previously described (Nakano et al., 2004). However, we did not observe transfer of the *bla*_IMP−1_ gene-encoding plasmid to either *E. coli* J53 or to *P. aeruginosa* PAO1.

## Discussion

*C. thiooxydans* is prevalent in the natural environment; however, it can also cause infection and/or colonization in human clinical settings. *C. thiooxydans* producing IMP-8 has been reported from China by Guo et al. (2021) with IMP-8 encoded by a chromosomal gene. *C. thiooxydans* NR4028 possessed plasmid-encoded IMP-1, a common carbapenemase gene in Japan, and resistanct to meropenem.

Figure 1 indicades the comparison of the plasmid structure of *C. thiooxydans* NR4028 with similar plasmid replicase genes and the genetic environment of resistance genes in IMP-1-encoded plasmids. The plasmid *repA* replicase gene that was present in this isolate was different from that circulating in Enterobacterales, but had high homology to those of plasmids in non-fermenting gram-negative bacteria, including *Vitreoscilla filiformis* (CP022425), *Xanthomonas* spp. (CP024031), and *P. aeruginosa* (KR106190) (Bi et al., 2016) (Figure 1A). Therefore, IMP-1 may spread among the less common bacterial species, as mentioned above. We found a genetic structure of this plasmid and genetic environment that are different from those previously described (Wajima et al., 2020; Mori et al., 2021). IMP-type carbapenemase genes are frequently located in class 1 integrons; however, in this case, IS*26* was located upstream of *bla*_IMP−1_ (Figure 1B). It is possible that the integrase gene was lost as a consequence of the insertion of the IS element.

This plasmid pNR4028_IMP1 was found to have a unique genetic structure. The IMP-1-encoding plasmid was about 30 kbp in length and did not have a locus Tra region, which is an essential region for conjugation. Due to the lost of the locus Tra region in this plasmid, it is suggested that the IMP-1-encoding plasmid could not be transferred to recipients by conjugation. However, it is necessary to characterize this unique plasmid by conducting transformation experiments with the plasmid or cloning the resistance genes, including IS*26* in the future.

In conclusion, to the best of our knowledge, this is the first report of plasmid-encoded IMP-1 producing *C. thiooxydans*. This plasmid has a unique structure; therefore, the dissemination of both this species and this plasmid should be monitored.

## Author Contributions

YS: conceptualization, methodology, investigation, and writing of the original draft. RN: critical revision. AN, HT, TA, SH, KS, MW, YNo, DK, S-TL, KU, AK, YNi, RS, MOg, MOh, and TS: validation and data curation. KT, KK, and HY: supervision and project administration. All authors contributed to the manuscript and approved the submitted version.

## Funding

This study was supported by JSPS KAKENHI grant 21K17897.

## Conflict of Interest

RS and MOg are employed by BML Inc. The remaining authors declare that the research was conducted in the absence of any commercial or financial relationships that could be construed as a potential conflict of interest.

## Publisher's Note

All claims expressed in this article are solely those of the authors and do not necessarily represent those of their affiliated organizations, or those of the publisher, the editors and the reviewers. Any product that may be evaluated in this article, or claim that may be made by its manufacturer, is not guaranteed or endorsed by the publisher.
